# Academic Impact of Qualitative Studies in Healthcare: Bibliometric Analysis

**DOI:** 10.1371/journal.pone.0057371

**Published:** 2013-03-13

**Authors:** Hiroko Mori, Takeo Nakayama

**Affiliations:** 1 Department of Health Informatics, School of Public Health, Kyoto University, Kyoto, Japan; 2 Department of International and Cultural Studies, Tsuda College, Kodaira-shi, Tokyo, Japan; University of Warwick, United Kingdom

## Abstract

**Context:**

Although qualitative studies are becoming more appreciated in healthcare, the number of publications of quality studies remains low. Little is known about the frequency and characteristics of citation in qualitative studies.

**Objective:**

To compare the academic impact of qualitative studies to that of two quantitative studies: systematic reviews and randomized controlled trials.

**Methods:**

Publications in BMJ between 1997 and 2006 (BMJ’s median impact factor was 7.04 during this period) employing qualitative methods were matched to two quantitative studies appearing the same year using PubMed. Using Web of Science, citations within a 24-month publication period were determined. Additionally, three hypotheses were examined: qualitative studies are 1) infrequently cited in original articles or reviews; 2) rarely cited by authors in non-English-speaking countries; and 3) more frequently cited in non-medical disciplines (e.g., psychology or sociology).

**Results:**

A total of 121 qualitative studies, 270 systematic reviews, and 515 randomised controlled trials were retrieved. Qualitative studies were cited a total of 1,089 times, with a median of 7.00 times (range, 0–34) for each study. Matched systematic reviews and randomized controlled trials were cited 2,411times and 1,600 times, respectively. With respect to citing documents, original articles and reviews exceeded 60% for each study design. Relative to quantitative studies, qualitative studies were cited more often by authors in English-speaking countries. With respect to subject area, medical disciplines were more frequently cited than non-medical disciplines for all three study designs (>80%).

**Conclusion:**

The median number of citations for qualitative studies was almost the same as the median of BMJ’s impact factor during the survey period. For a suitable evaluation of qualitative studies in healthcare, it will be necessary to develop a reporting framework and include explicit discussions of clinical implications when reporting findings. Coordination between researchers and editors will be needed to achieve this goal.

## Introduction

The importance of qualitative studies is becoming recognized in the healthcare research field. Although qualitative studies are often defined differently and inconsistently, the Medical Subject Headings of PubMed refer to them as” research that derives data from observation, interviews, or verbal interactions and focuses on the meanings and interpretations of the participants”[1]. Qualitative research questions are devised by isolating subjective ideas, experiences, and values within a specific population[2]. Such studies aim to answer questions that are difficult to approach quantitatively, such as “what is X,” or *how* and *why* question [3].These questions lead to the generation of concepts and construction of model to explain phenomena using the generated concepts. For instance, some qualitative studies describe relationships between dying patients and their caregivers in end-of life care [4], or describe recognizing and responding to a suicidal crisis within a family[5]. Although qualitative studies may be considered mysterious[6] or a “secondary science” by conservative quantitative researchers, their importance is underscored by the publication of editorials and methodological papers in leading healthcare journals [3], [7]. Despite this, a previous study reported that the prevalence of qualitative studies in healthcare journals remains low [8]. This may reflect the fact that qualitative studies have not yet been placed in the context of evidence-based medicine, and thus are not ranked in the hierarchy of evidence models[9].

One objective method used to gauge the academic impact of a research study is citation analysis. This method calculates the number of times a particular research article is cited in other papers to quantify its influence. Despite its limitations [10], [11], [12], this method has been popular among researchers as an objective way to evaluate research article [13], [14]. Patsopulos et al. compared the number of citations for various study designs[15], and reported a positive correlation between the hierarchy of evidence and citation frequency. However, this analysis did not include qualitative studies.

To evaluate academic impact quantitatively, we determined the citation pattern of qualitative studies compared with systematic reviews (SRs) and randomized controlled trials (RCTs). SR is defined in the Cochrane Handbook as“a review of a clearly formulated question that uses systematic and explicit methods to identify, select, and critically appraise relevant research, and to collect and analyze data from the studies that are included in the review.” [16]. RCTs are defined as” an experiment in which two or more interventions, possibly including a control intervention or no intervention, are compared by being randomly allocated to participants. In most trials one intervention is assigned to each individual but sometimes assignment is to defined groups of individuals (for example, in a household) or interventions are assigned within individuals (for example, in different orders or to different parts of the body)”[16] .

Our primary purpose was to determine and compare citation frequencies for research articles corresponding to these three study designs. To characterize the citation patterns of qualitative studies further, the following hypotheses were set forth: 1) qualitative studies are more frequently cited in editorials or letters expressing expert opinions compared with other document types (e.g., original articles); 2) given language barriers, qualitative studies that use descriptive data and are published in English are rarely cited by authors in non-English-speaking countries; and 3) qualitative studies are more frequently cited in non-medical disciplines (e.g., psychology or sociology) compared with SRs or RCTs.

## Methods

### Study selection

**Figure 1:**
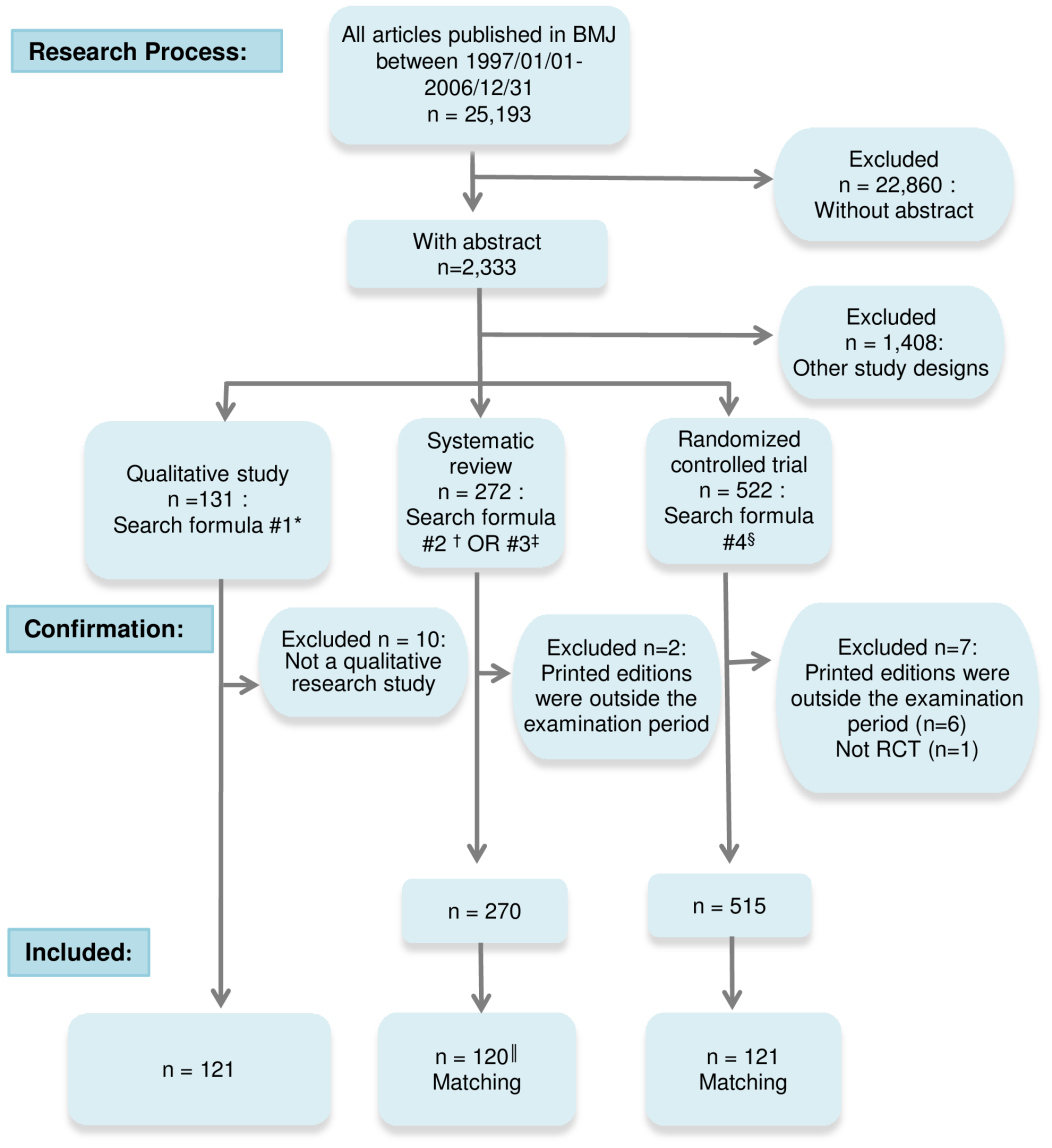
Selection procedure for studies surveyed in each study design. *Search formula # 1: "BMJ[jour] AND (qualitative AND (research OR study); Limits: only items with abstracts, Publication Date from 1997/1/1 to 2006/12/31". †Search formula # 2: "BMJ[jour] Limits: Meta-Analysis Limits: only items with abstracts, Publication Date from 1997/1/1 to 2006/12/31". ‡ Search formula # 3: "BMJ[jour] AND ("systematic review"[ti]) Limits: only items with abstracts, Publication Date from 1997/1/1 to 2006/12/31". §Search formula # 4: "BMJ Limits: only items with abstracts, Randomized Controlled Trial, Publication Date from 1997/1/1 to 2006/12/31". ||Although we identified 15 qualitative studies in 2001, we only confirmed 14 systematic reviews in that year. Therefore, the sum of cited documents for this type of publication was 120.

We surveyed articles that used qualitative methodologies and complete abstracts published in the printed edition of the British Medical Journal (BMJ) between January 1, 1997 and December 31, 2006. The scope of our survey was limited to BMJ not only because it publishes SRs, RCTs, and qualitative studies, but also because it has a well-established checklist for qualitative studies[17]. BMJ’s impact factor (IF) in 2011 was the sixth highest among 155 general medical journals as determined by Journal Citation Reports (JCR). Given that publications in high IF journals are cited often, we considered BMJ a journal suitable for our study purpose. To identify qualitative studies, an electronic search on PubMed was performed using the following keywords: *qualitative*, *research*, and *study*. The abstracts were then subsequently reviewed to confirm the use of qualitative methods. We used the “Limits” function and “Type of Article” search field to identify SRs published in BMJ listed as “Meta-Analysis,” and for those using the term “systematic review” in the study title. In addition, we used the “Limits” function and “Type of Article” search field to identify articles published in BMJ listed as “Randomized Control Trials.” A single SR and RCT article was matched to a qualitative study published in the same year. When multiple matching candidate SRs or RCTs were identified, articles were selected randomly and the number of qualitative studies, SRs, and RCTs were equalized for each year. Figure 1 summarizes the entire research process.

### Primary outcome: Citations in a 24-month period

The total number of times an article was cited during a 24-month period following publication (24-month citations), was the main outcome. This outcome measure is analogous to the concept of *impact factor*, which is an indicator of a journal’s influence based on citation frequency. The Web of Science (WOS) database maintained by Thomson Reuters was used to obtain data regarding citation frequency.

Although BMJ is published weekly, some of the citing documents analysed were published monthly or annually. For these documents, the date corresponding to the middle of the month or year of publication was used to determine whether certain articles would be included in the post-publication survey period. For example, if a particular document published in May 2002 cites two studies from BMJ that published on May 6, 2000 and May 27, 2000, the cut-off date for inclusion in the 24-month citation period would be May 6, 2002 and May 27, 2002, respectively. The publication date for this citing document would be considered May 15, 2002. Therefore, the study published on May 6 would be excluded, while that published on May 27 would be included in the 24-month citations data.

### Characteristics of citing documents

The three hypotheses were tested using the WOS function for citation analysis. To test the first hypothesis, the citing document was classified into one of 10 document types and the number of citations was determined using the WOS analysis function. This function classifies retrieved publications by their characteristics, for example, funding, the author affiliations, publication year. The 10 document types were articles (original contributions and not meta-analyses), reviews, editorial materials, letters, meeting abstracts, news items, proceedings, reprints, book reviews, and corrections. With respect to the number of articles and document types, the number of qualitative study citations, SRs, and RCTs were calculated, and odds ratios (ORs) with 95% confidence intervals (95% CIs) were determined. OR is defined as “the ratio of the odds of an event occurring in one group to the odds of it occurring in another group [18]. Odds is a ratio of the number of people incurring an event to the number of people who do not have an event ” [19].The same analysis was performed for the number of reviews and document types. Data were then used to compare citation patterns of qualitative studies with those of SRs and RCTs. All statistical analysis was performed using SPSS Version 17.0 (SPSS Inc, Chicago, IL).

To evaluate the relationship between language barrier and citation frequencies, the location of the research institution corresponding to the citing author was identified using WOS analysis function. These locations were then classified into English-speaking or non-English-speaking countries using the World Factbook[20] from the Central Intelligence Agency of the United States and Anglosphere from Wikipedia[21]. Instances where the author belonged to a research institution in a non-English-speaking country, but was studying abroad (e.g., the United States) at the time of publication or affiliated with research institutions in both English-speaking and non-English-speaking countries, were counted once. ORs comparing qualitative studies with SRs and RCTs were calculated.

To test the third hypothesis, citation frequencies for citing documents were determined across subject areas and classified into medically or non-medically relevant groups using the WOS index. Subject areas that were difficult to discriminate (e.g., social sciences and biomedical) were classified into the non-medical group. There were cases in which a single journal covered multiple academic areas (e.g., developmental psychology or educational psychology). In such cases, the total number of journal subject areas was greater than the total number of citations. Citation frequencies corresponding to academic subject areas were determined, and the 10 subject areas in which citations appeared with higher frequency were reported.

### Secondary outcome: cumulative citations

Citations of a research paper were found to increase during the first two or three years after publication, and decrease thereafter. This phenomenon is referred to as “citation dynamics.” “24-month citations,” i.e., the primary outcome of this study, reflects the peak number of citations during this period. To investigate the effects of citation dynamics, we analyzed cumulative citations (i.e., the total number of citations from publication to the time of this study (September 8 to October 6, 2009)) for the three study designs. Moreover, the three hypotheses described above were examined with respect to cumulative citations in a manner similar to that for 24-month citations.

## Results

### Surveyed Studies

The PubMed search was conducted on February 5, 2009 and a total of 25,193 documents (including 2,333 abstracts) from January 1, 1997 to December 31, 2006 were examined. Of these documents, 131 were qualitative study articles and 121 satisfied the inclusion criteria. Likewise, 272 SR articles were identified. However, two publications were excluded as these were outside the examination period, thereby resulting in 270 SR articles. A total of 522 RCT articles were identified, six of which were excluded as these were outside the examination period. An additional article was excluded because it was classified incorrectly, bringing the total number of RCT articles to 515. BMJ’s median impact factor during the surveyed period was 7.04 (range: 9.72–4.99). The number of qualitative studies, SRs, and RCTs during the surveyed periods comprised 38.8 % of BMJ’s publications with abstract. A single SR and RCT article was matched with each of the 121 qualitative studies. However, it was not possible to match one of the SR articles with the 15 qualitative studies published in 2001, as only 14 SR articles were published that year. Therefore, the total number of SRs surveyed was 120 (Figure 1).

### Number of citations and characteristics

Data corresponding to 24-month citations were collected during September 8 and October 6, 2009. Qualitative studies were cited 1,089 times (median 7, range 0–34), SRs were cited 2,411 times (median 14, range 0–88), and RCTs were cited 1,600 times (median 10, range 2–67; Table 1).

**Table 1:** Citing Document Types.

	Citation counts				Document type									
	N	Median (min-max)	IQR*	P value	Article† (%)	Review (%)	Editorial Material (%)	Letter (%)	Meeting abstract (%)	News item (%)	Proceeding paper (%)	Reprint (%)	Book review	Correction
Qual‡	1089	7.0 (0–34)	8	Reference	632 (58.0)	84 (7.7)	166 (15.2)	145 (13.3)	1 (0.1)	10 (0.9)	48 (4.4)	2 (0.2)	0 (n/a)	1 (0.1)
SR‡	2411	14.0 (0–88)	19	0.00	1209 (50.1)	462 (19.2)	311 (12.9)	248 (10.3)	4 (0.2)	16 (0.7)	150 (6.2)	2 (0.1)	0 (n/a)	9 (0.4)
RCT‡	1600	10.0 (2–67)	12	0.00	825 (51.6)	267 (16.7)	181 (11.3)	205 (12.8)	4 (0.3)	17 (1.1)	93 (5.8)	2 (0.1)	1 (0.1)	5 (0.3)

	Articles vs. other document types				Reviews vs. other document types			
	Article	Others	OR (95% CI)		Review	Others	OR(95% CI)	
Qual‡	632	457	1.37 (1.19–1.59)	1.31 (1.12–1.53)	84	1005	0.35 (0.28–0.45)	0.41(0.32–0.54)
SR‡	1209	1202	Reference	―	462	1949	Reference	―
RCT‡	825	775	―	Reference	267	1333	―	Reference

* IQR was Inter Quartile Range

† Articles considered were only original contributions and not meta-analyses. Definitions of document types were determined by WOS.

‡ Qual: qualitative study, SR: systematic review, RCT: randomized controlled trial

Articles were the most common of the 10 document types, comprising 58.0% (632/1089) of all documents that cited the 121 qualitative studies, 50.1% (1209/2411) of all documents citing SRs, and 51.6% (825/1600) of all documents citing RCTs (Table 1). Article citations were compared between qualitative studies and SRs or RCTs, and ORs were calculated. ORs obtained for the comparison to SRs and RCTs were 1.37 (95% CI, 1.19–1.59) and 1.31 (95% CI, 1.12–1.53), respectively (Table 1). With respect to qualitative studies, the most common document type was editorials (15.2%, 166/1089), followed by letters (13.3 %, 145/1089) and reviews (7.7%, 84/1089). With respect to SRs and RCTs, the most common document type was reviews (SRs: 19.2%, 462/2411; RCTs: 16.7%, 267/1600; Table 1). A comparison of citation frequencies for reviews resulted in ORs of 0.35 (95% CI, 0.28–0.45) and 0.41 (95% CI, 0.32–0.54) relative to SRs and RCTs, respectively (Table 1). The proportion of editorial material among citing documents was 15.2% for qualitative studies, 12.9% for SRs, and 11.3% for RCTs (Table 1). The proportion of letters among citing documents was 13.3% for qualitative studies, 10.3% for SRs, and 12.8% for RCTs.

To test the second hypothesis, we examined how each study was cited by researchers in non-English-speaking countries. A comparison between qualitative studies and SRs resulted in an OR of 2.06 (95% CI, 1.75–2.41). A comparison between qualitative studies and RCTs resulted in an OR of 1.69 (95% CI, 1.42–2.00; Table 2).

**Table 2:** Citation frequencies of articles published in English-speaking and non-English-speaking countries.

		English-speaking	non-English-speaking	OR (95% CI)	
24-month citations					
	Qual*	945	252	2.06 (1.75–2.41)	1.69 (1.42–2.00)
	SR*	1888	1035	Reference	―
	RCT*	1260	567	―	Reference

* Qual: qualitative study, SR: systematic review, and RCT: randomized controlled trial.

The number of citing documents across all subject areas was 1,504, 3,277, and 2,197 for qualitative studies, SRs, and RCTs, respectively. Medically relevant areas, which included nursing and pharmacology, accounted for over 80% of citations in each of the three study designs. Table 3 summarizes the 10 academic subject areas in which qualitative studies, SRs, and RCTs were cited. The subject area, “Medicine, General and Internal,” had the highest number of citations in each study design.

**Table 3:** Corresponding subject areas for citing documents and 24-month citation frequencies.

	QUAL *			SR *			RCT *		
Subject Area+	No. of citations	Ranking	Relative cumulative frequency	No. of citations	Ranking	Relative cumulative frequency	No. of citations	Ranking	Relative cumulative frequency
MEDICINE, GENERAL & INTERNAL	462	1	30.7%	760	1	23.2%	550	1	25.0%
HEALTH CARE SCIENCES & SERVICES	126	2	39.1%	95	7	49.6%	68	7	49.7%
PUBLIC, ENVIRONMENTAL & OCCUPATIONAL HEALTH	122	3	47.2%	189	3	35.3%	88	4	39.6%
ONCOLOGY	61	4	51.2%						
SOCIAL SCIENCES, BIOMEDICAL	45	5	57.2%						
MEDICAL INFORMATICS	45	6	57.2%						
PSYCHIATRY	43	7	60.1%	86	8	52.2%	108	3	35.6%
PHARMACOLOGY & PHARMACY	37	8	62.5%	209	2	29.6%	124	2	30.7%
EDUCATION, SCIENTIFIC DISCIPLINES	31	9	64.6%						
CLINICAL NEUROLOGY	27	10	66.4%				73	6	46.6%
CARDIAC & CARDIOVASCULAR SYSTEMS				145	4	39.8%	63	8	52.6%
GASTROENTEROLOGY & HEPATOLOGY				120	5	43.4%			
RESPIRATORY SYSTEM				106	6	46.7%	82	5	43.3%
PERIPHERAL VASCULAR DISEASE				85	9	54.8%			
PEDIATRICS							47	9	54.7%
GERIATRICS & GERONTOLOGY							47	9	54.7%
OBSTETRICS & GYNECOLOGY				82	10	59.8%			

* Qual: qualitative study, SR: systematic review, RCT: randomized controlled trial.?Subject areas were categorized using the WOS function.

### Secondary outcome: cumulative citations

With respect to cumulative citations, qualitative studies were cited 5,147 times, SRs 12,111times, and RCTs 7,286 times. Compared to qualitative studies, cumulative citations for SRs and RCTs were 1.4 - and 2.4 -fold higher, respectively. With respect to document types, original articles and reviews comprised 80% of citing documents for all study designs. Regarding the second hypothesis, a comparison of cumulative citations between qualitative studies and SRs and RCTs resulted in ORs of 1.74 (95% CI, 1.63–1.85) and 1.45 (95% CI, 1.36–1.55), respectively. With respect to subject area, medical disciplines were more frequently cited than non-medical disciplines for all three study designs (>80%).

### Reproducibility

Among surveyed studies, all qualitative studies were included, while SRs and RCTs were randomly selected. To test the reproducibility with random selection and matching, 24-month citations for two other datasets which each consisted of 121 randomly selected RCTs from the 515 RCTs were calculated. In the first data set, the RCTs were not matched by year. In the second dataset, RCTs were matched by year. The 24-month citations for the first dataset was 1,664 (median, 11; range, 1–71), and the 24-month citations for the second dataset was 1,624 (median, 10.0; range, 1–127). This result did not significantly differ from our original results.

## Discussion

To our knowledge, this is the first study to examine citation frequency and the characteristics of qualitative studies and compares citation patterns of qualitative studies with those of quantitative studies. Although qualitative studies are cited less frequently than SRs and RCTs, the median of 7 for 24-month citations for qualitative studies was almost the same as the median of the impact factor of the journal, BMJ, from which the surveyed articles were derived. Based on our results, qualitative studies can be considered to have appreciated citations.

Previous reports have shown impact factors for papers according to research funding[22], [23] , sample size[22], [24] , clinical discipline[22], [25] , number of authors [22], [26] and nationality[27] , journal impact factor, and study design[22], [28] . However, no reports have been published on qualitative studies. With respect to qualitative studies in healthcare, these characteristics, share of publications [29], [30] , and journal nationality [8], [31] have been reported, but the characteristics on citations of qualitative studies, compared with other study designs, has not. In the present study, we found that the citation frequency of qualitative studies was approximately half and two-thirds compared with SRs and RCTs, respectively, and that qualitative studies have an academic impact similar to SRs and RCTs.

The present study also tested three hypotheses to characterize citation patterns of qualitative studies. The first hypothesis, however, was not supported. In fact, articles were the document type most frequently cited in all three study designs. ORs obtained from our analysis indicated that qualitative studies had a higher frequency compared with SRs and RCTs. In contrast, qualitative studies were cited at a lower frequency in reviews compared with SRs and RCTs. One possible explanation for this observation is that qualitative studies do not contribute to meta-analyses as a secondary source of data. The second hypothesis regarding potential language barriers and the citation of qualitative studies was supported. Our results show a lower frequency of citations for qualitative studies in papers published in non-English-speaking countries. Numerical data where SRs and RCTs were indicated have greater adaptability and high relevance for similar research questions. In contrast, qualitative data where qualitative studies were indicated in order to answer “what is X,” or how and why questions, offered important insights. Properties of qualitative data tend to segregate in distinct generality compared with those of numerical data. In addition, when a research project name, which consists of multiple researchers with multiple affiliations, is enrolled as the author of the citing document in the WOS database, the locations of research institutions are usually missing when using the WOS function. A total of 24 documents were missing data among 1,089 citing documents for qualitative studies, 70 documents among 2,411citing documents for SRs, and 57 documents among 1,600 citing documents for RCTs. These documents with missing data, however, did not affect the results obtained while addressing the second hypothesis. Finally, our results did not support our third hypothesis. Qualitative studies were cited primarily in medically relevant areas, similar to SRs and RCTs. Moreover, with respect to cumulative citations, citation dynamics did not have an effect on the results of this study.

There are four limitations to our study worth noting. First, the present study used only one database. Additional databases exist (e.g., SciVerse Scopus); yet, we feel there is little merit to using multiple databases in this analysis when considering the objectives. Second, properties obtained using the WOS database were used. Due to the location of the research institution and the academic discipline of the journals in which the citing documents were published, multiple data points were allotted to a single citing document. Third, while there are other influential medical journals (e.g., *JAMA*, *the Lancet*, and *New England Journal of Medicine: NEJM*), only studies published in BMJ were surveyed. However, the total number of qualitative studies for *JAMA*, *the LANCET* and *NEJM* during the surveyed period were 5/3299(0.015%), 10/4678 (0.002), and 0/2199(0%), respectively. Focusing our analysis to studies published in BMJ was an important choice given the journal’s pioneering role in publishing qualitative studies, thereby ensuring the internal validity of our study. Fourth, although all published qualitative studies were included in this survey, SRs and RCTs were randomly sampled and matched. To address this, we tested reproducibility based on two other datasets of 121 RCTs from 515 RCTs by random selections, and confirmed that no significant biases were existed.

The academic impact of qualitative studies in healthcare may have been underestimated thus far. Our present findings lay the basis for proposals to editors and researchers in healthcare fields such that qualitative studies are evaluated fairly.

First, it is vital to develop reporting frameworks for qualitative studies that facilitate the conveyance of validity, social context, and research methods within the framework of limited manuscript length. By nature, qualitative study reports are descriptive. Just as the CONSORT statement has improved the reporting of RCTs [32], [33], qualitative descriptions need to be structured to increase scientific validity. Although a number of checklists exist, such as Consolidated Criteria for Reporting Qualitative Research (COREQ) developed by researchers and BMJ’s criteria developed by editors, a collaborative effort between researchers and editors may serve to establish guidelines similar to the CONSORT statement for reporting qualitative studies[17], [34]. Second, when reporting, authors should more explicitly state the clinical implications of their findings. Indeed, in addition to explaining social phenomena, qualitative studies in healthcare must also aim to promote human health. Although generally not intended to be as generalizable as the results of quantitative studies, findings in qualitative studies can be transferable to other contexts and readers can assess whether they are applicable to their own settings or not [35]. Such findings can help clinicians grasp the complicated nature of reality, and may prompt researchers to conduct further studies. Transferability and applicability, two peculiar properties of qualitative studies, could realize these clinical implications. During the process of reviewing a manuscript for publication, it is expected that editors and reviewers will prompt authors to explicitly indicate any clinical implications of their findings.

This study focused on the citation frequency and characteristics of qualitative studies. To solve diverse issues in health science and clinical practice, cooperation between qualitative researchers, quantitative researchers, clinicians, and editors will be important. In particular, it is anticipated that the leadership of editors in this process will help propel healthcare forward.
